# Stochastic Dynamics of Nonlinear Piezoelectric Vibration Energy Harvesting System with Inelastic Impact

**DOI:** 10.3390/e28040400

**Published:** 2026-04-01

**Authors:** Li Liu, Lili Tian, Meng Su, Hongge Yue

**Affiliations:** 1 School of Mathematics and Statistics, Ningxia University, Yinchuan 750021, China; 2 School of Mathematics, Northwest University, Xi’an 710127, China

**Keywords:** energy harvesting system, inelastic impact, generalized stochastic averaging method, stochastic response, stochastic stability

## Abstract

Because the introduction of a vibro-impact structure can widen the bandwidth and improve the harvesting efficiency of the vibration energy harvesting (VEH) systems, an analytical method for a VEH system based on vibro-impact is proposed to employ the stochastic response and stability. Firstly, the piezoelectric control equation is decoupled by the generalized harmonic transformation, which obtains an uncoupled equivalent system. Secondly, the Itô stochastic differential equation with amplitude is analytically derived by applying the proposed analytical method. Furthermore, the influence of crucial parameters on the mean square voltage (MSV) and the mean output power is explored, such as the coupling factors and restitution coefficient. Finally, the top Lyapunov exponent (TLE) can be derived based on the linearized averaged Itô equations and the condition for the stability with probability one is obtained. It turned out that restitution coefficient *r* and time constant ratio μ have remarkable effects on the system’s stability.

## 1. Introduction

In recent years, energy harvesting systems have attracted considerable attention as a promising technology for powering wireless sensor networks, Internet of Things (IoT) devices, and wearable electronics [1]. These systems enable the extraction of energy from ambient sources and its conversion into usable electrical power, thereby offering the potential for self-sustainability and prolonged device operation without the need for external power sources. The primary methods of vibration energy harvesting encompass electromagnetic energy harvesting [2,3], piezoelectric energy harvesting [4,5], and electrostatic energy harvesting [6]. Linear energy harvesting systems were introduced firstly to design the VEH, which have a narrow frequency bandwidth and limited applications; therefore, nonlinearity is adopted to solve these problems [7,8,9]. Nonlinear energy harvesting systems utilize emerging energy conversion mechanisms such as piezoelectric effects, magnetic coupling phenomena, and thermoelectric effect. In contrast to linear energy collectors, nonlinear vibration energy collectors can collect vibration energy more effectively in a wide frequency range, so it is more suitable for vibration energy collection in real life. Consequently, the design and development of nonlinear vibration energy harvesters have attracted substantial research interest. Erturk [7] developed a non-resonant piezomagnetoelastic energy harvester to address the narrow bandwidth limitation inherent in conventional resonant cantilever designs. Daqaq [8] demonstrated that hardening stiffness reduces the amplitude at resonance and induces an upshift in the resonant frequency. Stanton et al. [9] developed a harvester with a single resonant mode exhibiting nonlinear behavior that extends the bandwidth of effective energy conversion by modulating the magnetic force, thereby enabling the harvester to exhibit either soft or hard nonlinear characteristics. Yue [10] adopted the generalized cell mapping methodology to characterize the global dynamical behaviors of VEH systems. For real-world engineering applications, energy harvesting systems must operate under randomly varying environmental influences and external noise disturbances. Therefore, it is necessary to consider the influence of random environment excitation on the performance of nonlinear energy harvesters. Consequently, it is attractive to establish analytical frameworks for investigating the performance of the stochastic. Daqaq [11] utilized the method of moments to analyze the voltage response statistics and revealed the influence of the time constant ratio on the performance enhancement of VEHs under Gaussian white noise excitation. Green [12] presented the performance of the energy harvester, which incorporates a magnetic levitation device to extend its bandwidth. Zhang [13] applied the improved stochastic averaging method to investigate the stochastic response and the performance analysis of VEHs under correlated colored noise. Xu applied the different stochastic averaging technique to study the stationary response of mono-stable nonlinear energy harvesters [14] and bistable nonlinear energy harvesters [15]. Jiang [16] applied the equivalent linearization technique to investigate the stochastic response and performance analysis about the nonlinear piezoelectric vibration energy harvesting (PVEH). The above-mentioned papers only consider the stochastic response and performance of PVEH; the stochastic dynamical behaviors of PVEH with nonlinear mechanical vibro-impact have not yet been studied.

Nonlinear mechanical vibro-impact is adopted in the application of PVEH technologies to extend the bandwidth and improve the harvesting efficiency, which was studied by some scholars in [17,18]. Some structurally sophisticated nonlinear vibrational energy harvesting (VEH) systems such as S-shaped [19] and X-shaped [20] ones have been developed to enhance energy conversion efficiency. Numerical responses for impact energy harvesting can still be acquired even when certain parameters are estimated through a simplified impact force model [21,22,23]. Halim [24] proposed that the PVEH system with a mechanical impact mechanism would increase output power and operating frequency bandwidth. Tyler [25] examined the potential of vibro-impact energy harvesters to enhance energy harvesting efficiency through a direct investigation of the asymmetric gaps at rigid barriers. Le [26] found that machine impacts are adopted to enlarge the output energy of the PVEH system. Jacquelin [27] found that impact can increase the energy harvestin performance. Lai [28] studied the dynamic and electrical behaviors of a vibro-impact wind energy harvester utilizing a dielectric elastomer (DE-based) generator. Cao [29] applied theoretical analysis and experimental tests to find that the VEH system with a vibro-impact structure produces a greater output voltage compared to non-impact types. Deng [30] proposed a novel U-shaped vibro-impact galloping energy harvester and further investigated its stochastic dynamic response. Huang [31] established the theoretical framework for a bio-inspired energy harvester utilizing a wing-beat pattern.

Additionally, stochastic stability is a critical factor in system safety, as serious consequences can arise once it is compromised. Meanwhile, the noise exists unavoidably and has a greatly significant role in the real system. Hence, stochastic stability has been extensively investigated. Compared with smooth systems, the study on the immature stability of the vibro-impact becomes more complex due to the discontinuity and complexity. Khasminskii [32] explored the expression of TLE about a linear stochastic differential equation. Kozin [33] established the necessary and sufficient conditions for stability in nonlinear systems. Zhu [34,35] explored the stochastic averaging method to investigate the stochastic Hamiltonian system based on Khasminskii theory. Lin [36] concentrated on investigating the response and the asymptotic stability with probability one of a strongly nonlinear viscoelastic system under wide-band noise. Liu [37,38] investigated the stochastic stability of the quasi-Hamiltonian systems under the parametric excitations of combined Gaussian and Poisson white noise. Qiao [39] investigated the stochastic asymptotic stability with probability one of a variable mass system. Su [40] investigated the stochastic response and stability of a linear vibro-impact system with friction. Prevailing studies predominantly address the deterministic models of energy harvesting systems and their stochastic performance under conditions of random excitation.

In contrast, few studies have addressed the stochastic behavior of energy harvesting systems that involve impact mechanisms, and few studies address the stochastic stability of impact-based energy harvesting systems. Therefore, it is imperative to investigate the stochastic response and stability of impact-driven energy harvesting systems under random excitation.

This paper mainly focuses on analyzing the stochastic response and the asymptotic Lyapunov stability with probability of the stochastic impact energy harvesting system. In Section 2, a nonlinear model is established for a piezoelectric vibration energy harvester (PVEH) that incorporates impact dynamics. The electromechanical coupled equations are decoupled by employing the generalized harmonic transformation method. In Section 3, we derive the Itô stochastic differential equation for the amplitude, from which the stationary response and the mean square value (MSV) are obtained. Additionally, the condition for asymptotic stability with probability one of the PVEH is investigated. The numerical results in Section 4 demonstrate the robustness and effectiveness of our theoretical approach. Meanwhile, the effects of some parameters of the system on the MSV and the asymptotic stability with probability one of the systems are explored.

## 2. System Description

A generalized model of a vibration energy harvester [11,41] featuring a unilateral classical inelastic barrier is introduced to broaden its operational bandwidth and enhance the energy conversion efficiency. This model consists of an inelastic baffle coupled to a piezoelectric harvesting system.

(1a) $$M\ddot{\bar{X}}+(b_{3}{\bar{X}}^{4}-b_{2}{\bar{X}}^{2}-b_{1})\dot{\bar{X}}+\delta_{1}\bar{X}+\delta_{2}{\bar{X}}^{3}+\bar{\beta }\bar{V}=-M{\ddot{\bar{X}}}_{b},\bar{X}>0,$$

(1b) $${\dot{\bar{X}}}_{+}=-r{\dot{\bar{X}}}_{-},\bar{X}=0,$$

(1c) $$C_{p}\dot{\bar{V}}+\frac{\bar{V}}{R_{V}}=\bar{\beta }\dot{\bar{X}}$$

In which $\bar{X}$ represents the displacement of the mass M and voltage $\bar{V}$ is measured across the equivalent resistive load $R_{V}$. Respectively, $b_{1},b_{2},b_{3}$ are damping coefficients. $C_{P},R_{V}$ represent respectively the piezoelectric capacitance and the load resistance. r represents the restitution coefficient. $\bar{\beta }$ represents the electromechanical coupling coefficient and ${\ddot{\bar{X}}}_{b}$ represents the base acceleration.

The equations of motion are nondimensionalized by applying the following transformations:

$$x=\frac{\bar{X}}{l},x_{b}=\frac{{\bar{X}}_{b}}{l},t=\omega_{0}\tau ,\omega_{0}=\sqrt{\frac{\delta_{1}}{M}},c_{3}=\frac{b_{3}l^{4}}{M\omega_{0}},c_{2}=\frac{b_{2}l^{2}}{M\omega_{0}},$$

$$c_{1}=\frac{b_{1}}{M\omega_{0}},\alpha =\frac{l^{2}\delta_{2}}{M{\omega_{0}}^{2}},\beta =\frac{{\bar{\beta }}^{2}}{M{\omega_{0}}^{2}C_{p}},\mu =\frac{1}{R_{V}C_{p}\omega_{0}},V=\frac{C_{p}\bar{V}}{\bar{\beta }l}$$

in which l represents a characteristic length scale (the ratio of the equivalent piezoelectric capacitor area to the inter-plate distance) and $\omega_{0}$ is the short-circuit natural frequency. Through the above transformations, Equation (1) is nondimensionalized and rewritten as follows:

(2a) $$\ddot{x}+(c_{3}x^{4}-c_{2}x^{2}-c_{1})\dot{x}+x+ax^{3}+\beta V=\varepsilon_{1}\xi_{1}(t)+\varepsilon_{2}x\xi_{2}(t),\;x>0,$$

(2b) $${\dot{x}}_{+}=-r{\dot{x}}_{-},\;x=0,$$

(2c) $$\dot{V}+\mu V=\dot{x},$$

where x represents the dimensionless displacement. $c_{1}$, $c_{3}$ and $c_{2}$ are the dimensionless damping coefficients, both linear and nonlinear. r(0<r<1) represents the restitution coefficient. ${\dot{x}}_{+}$ and ${\dot{x}}_{-}$ represent the instantaneous velocities before and after impact. β is the piezoelectric coupling coefficient; V and μ are the output voltage and the time constant ratio. Respectively, $\xi_{1}(t)$ and $\xi_{2}(t)$ are dependent Gaussian white noise with the following properties:

$$E\langle \xi_{i}(t)\rangle =0,\;R_{ij}(\tau )=E\langle \xi_{i}(t)\xi_{j}(t+\tau )\rangle =2D_{\mathrm{ij}}\delta (\tau )\;(i,j=1,2).$$

where $D_{11}$ and $D_{22}$ denote noise intensity and δ(τ) is Dirac function.

## 3. The Equivalent Nonlinear System

Using the generalized harmonic function, the system displacement and velocity can be expressed as

(3) $$\begin{array}{l} y(t)=A\cos\Phi (t),\\ \dot{y}(t)=-A\nu (A,\Phi )\sin\Phi (t),\\ \Phi (t)=\theta (t)+\Gamma (t),\\ \nu (A,\theta )=\frac{d\theta (t)}{dt}, \end{array}$$

in which the amplitude A and transient phase Γ(t) are slow-varying random processes. ν(A,Φ) is the instantaneous frequency.

By integrating Equation (2c), the voltage expressions can be derived.

$$V(t)=C(t)e^{-\mu t}+\int_{0}^{t}e^{-\mu (t-\tau )}\dot{x}(\tau )d\tau ,$$

Neglecting the term $C(t)e^{-\mu t}$ and employing the transformation s=t−τ, the above equation can be approximated as

(4) $$V(t)\approx \int_{0}^{t}e^{-\beta s}\dot{x}(t-s)d\tau ,$$

ω(A) is the average frequency; then, Φ(t) can be expressed using the following approximate formula:

Φ(t)=ω(A)t+Γ(t),

One can obtain the following approximated expression:

(5) $$\dot{x}(t-s)\approx -A\omega (A)\sin[\omega (A)(t-s)+\Gamma ]\approx \dot{x}(t)\cos[\omega (A)s]+x(t)\omega (A)\sin[\omega (A)s],$$

By neglecting the exponential decay term, the current and voltage can be obtained from Equation (5) through the substitution of Equation (4).

$$V(t)\approx \frac{A\omega (A)}{\beta^{2}+\omega^{2}(A)}(\omega (A)\cos\theta -\beta \sin\theta )$$

Equation (5) can be approximately obtained by substituting Equation (3):

(6) $$V(t)\approx \frac{\omega^{2}(A)}{\mu^{2}+\omega^{2}(A)}x+\frac{\mu }{\mu^{2}+\omega^{2}(A)}\dot{x},$$

Substituting Equation (6) into the mechanical equation in Equation (2a), the modified equation can be written as follows:

(7a) $$\begin{array}{l} \ddot{x}+\left(c_{3}x^{4}-c_{2}x^{2}-c_{1}+\frac{\beta \mu }{\mu^{2}+\nu^{2}(A,\Phi )}\right)\dot{x}+\left(1+\frac{\beta \nu^{2}(A,\Phi )}{\mu^{2}+\nu^{2}(A,\Phi )}\right)x+\alpha x^{3}\\ =\varepsilon_{1}\xi_{1}(t)+\varepsilon_{2}x\xi_{2}(t),\;x>0, \end{array}$$

(7b) $${\dot{x}}_{+}=-r{\dot{x}}_{-},\;x=0.$$

The frequency function can be expressed by potential energy:

(8) $$\nu (A,\Phi )=\frac{d\theta }{dt}=\sqrt{\frac{2[G(A)-G(A\cos\Phi )]}{A^{2}\sin^{2}\Phi }}.$$

in which potential function G is given as

$$G=\int_{0}^{x}g(u)du,$$

$$g(x)=\left(1+\frac{\beta \omega^{2}(A)}{\mu^{2}+\nu^{2}(A)}\right)x+\alpha x^{3}.$$

The frequency function can be derived via the above Equation (8):

(9) $$\nu (A,\Phi )=\sqrt{\frac{\Gamma_{1}+\sqrt{\Gamma_{1}^{2}+4\Gamma_{0}}}{2}},$$

$$\Gamma_{1}=1+\beta +\frac{3}{4}\alpha A^{2}+\frac{1}{4}\alpha A^{2}\cos2\Phi -\mu^{2},$$

$$\Gamma_{0}=\mu^{2}+\frac{3}{4}\alpha A^{2}\mu^{2}+\frac{1}{4}\alpha A^{2}\cos2\Phi .$$

Thus, integrating Equation (9) with respect to Φ(t) over the interval from 0 to 2π yields the following approximate expression for the averaged frequency:

(10) $$\omega (A)=\sqrt{1+\beta +\alpha A^{2}}.$$

## 4. Non-Smooth Transformation and Stochastic Averaging Procedure

As proposed by Zhuravlev [42], the implementation of the non-smooth transformation for response displacement and velocity proceeds as follows:

(11) $$x=\left|y\right|=y\mathrm{sgn}(y),\;\dot{x}=\dot{y}\mathrm{sgn}(y),\;\ddot{x}=\ddot{y}\mathrm{sgn}(y).$$

where

sgn(y)=−1,  y<0; sgn(y)= 0, y=0; sgn(y)= 1  y>0.

It can be found that the transformation of Equation (11) maps the domain *x* > 0 of the original plane $\left(x,\dot{x}\right)$ onto the whole phase plane $\left(y,\dot{y}\right)$. The transformed equations of the new variables can be written by substituting Equation (11) into Equation (7a,b) as follows:

(12a) $$\begin{array}{l} \ddot{y}+\left(c_{3}y^{4}-c_{2}y^{2}-c_{1}+\frac{\beta \mu }{\mu^{2}+\omega^{2}(A)}\right)\dot{y}+\left(1+\frac{\beta \omega^{2}(A)}{\mu^{2}+\omega^{2}(A)}\right)y+\alpha y^{3}\\ =\varepsilon_{1}\mathrm{sgn}(y)\xi_{1}(t)+\varepsilon_{2}y\xi_{2}(t),\;t\neq t_{*}, \end{array}$$

(12b) $${\dot{y}}_{+}=-r{\dot{y}}_{-},\;t=t_{*}.$$

In the transformation, $t_{*}$ is the instant of impacts, which is not determined in advance. The jump of converted velocity $\dot{y}$ becomes proportional (1 − *r*) instead of (1 + *r*) for the original $\dot{x}$. By introducing Dirac delta function $\delta (t-t_{*})=\left|\dot{y}(t_{*})\right|\delta (y(t_{*}))$, Equation (12b) can be interpreted as introducing an additional impulse damping effect to Equation (12a) at each impact instance within a period, where the impulsive term is given by

(13) $$({\dot{y}}_{-}-{\dot{y}}_{+})\delta (t-t_{*})\approx (1-r)\dot{y}\left|\dot{y}\right|\delta (y).$$

Substituting Equation (11) and Equation (13) into system (12) yields the following approximate system:

(14) $$\begin{array}{l} \ddot{y}+(f(y)+C(A))\dot{y}+(1+K(A))y+\alpha y^{3}+(1-r)\dot{y}\left|\dot{y}\right|\delta (y)\\ =\varepsilon_{1}\mathrm{sgn}(y)\xi_{1}(t)+\varepsilon_{2}y\xi_{2}(t). \end{array}$$

in which

$$C(A)=\frac{\beta \mu }{\mu^{2}+\omega^{2}(A)},\;K(A)=\frac{\beta \omega^{2}(A)}{\mu^{2}+\omega^{2}(A)}.$$

The total energy *H* of the system (14) is given by the following expression:

$H=\frac{1}{2}\dot{y}+G(y),\;G(y)=\frac{1}{2}({\omega_{0}}^{2}+K(A))y^{2}+\frac{1}{4}\alpha y^{4}.$ Assuming that the periodic solution of system (14) takes the form of Equation (3), we have instantaneous frequency:

(15) $$\nu (A,\Phi )=\sqrt{\left(1+K(A)+\frac{3}{4}\alpha A^{2}\right)(1+\eta \cos2\Phi )}.$$

in which cosΦ(t) and sinΦ(t) are called the generalized harmonic functions. A(t) and Γ(t) are random processes. The instantaneous frequency ν(A,Φ) of the oscillation can be approximated by the following finite sum with a relative error less than 0.03%:

(16) $$\nu (A,\Phi )=b_{0}+b_{2}\cos2\Phi +b_{4}\cos4\Phi +b_{6}\cos6\Phi ,$$

in which

(17) $$\begin{array}{l} b_{0}={\left(1+K(A)+3\alpha A^{2}/4\right)}^{1/2}(1-\eta^{2}/16),\\ b_{2}={\left(1+K(A)+3\alpha A^{2}/4\right)}^{1/2}(\eta /2+3\eta^{3}/64),\\ b_{4}={\left(1+K(A)+3\alpha A^{2}/4\right)}^{1/2}(-\eta^{2}/16),\\ b_{6}={\left(1+K(A)+3\alpha A^{2}/4\right)}^{1/2}(\eta^{3}/64),\\ \eta =\frac{1}{4}\alpha A^{2}/\left(1+K(A)+\frac{3}{4}\alpha A^{2}\right). \end{array}$$

Substituting Equation (3) into the system (14), one can yield the stochastic differential equations of the amplitude and the phase angle.

(18) $$\begin{array}{l} \frac{dA}{dt}=m_{1}(A,\Phi )+\sigma_{11}(A,\Phi )\xi_{1}(t)+\sigma_{12}(A,\Phi )\xi_{2}(t),\\ \frac{d\Gamma }{dt}=m_{2}(A,\Phi )+\sigma_{21}(A,\Phi )\xi_{1}(t)+\sigma_{22}(A,\Phi )\xi_{2}(t), \end{array}$$

in which

$$m_{1}=-\frac{A\nu^{2}(A,\Phi )\sin^{2}\Phi [f(A\cos\Phi )+C(A)]}{1+K(A)+\alpha A^{2}}-\frac{\left(1-r)A^{2}\nu^{3}(A,\Phi )\left|\sin^{3}\Phi \right|\delta (A\cos\Phi \right)}{1+K(A)+\alpha A^{2}},$$

$$m_{2}=-\frac{\nu (A,\Phi )\sin\Phi \cos\Phi [f(A\cos\Phi )+C(A)]}{1+K(A)+\alpha A^{2}}-\frac{\left(1-r)A\nu^{3}(A,\Phi )\sin^{2}\Phi \cos\Phi \delta (A\cos\Phi \right)}{1+K(A)+\alpha A^{2}},$$

$$\sigma_{11}=-\frac{\varepsilon_{2}\mathrm{sgn}(A\cos\Phi )\nu (A,\Phi )\sin\Phi }{1+K(A)+\alpha A^{2}},\;\sigma_{12}=-\frac{\varepsilon_{2}A\nu (A,\Phi )\sin\Phi \cos\Phi }{1+K(A)+\alpha A^{2}},$$

(19) $$\sigma_{21}=-\frac{\varepsilon_{2}\mathrm{sgn}(A\cos\Phi )\nu (A,\Phi )\cos\Phi }{A(1+K(A)+\alpha A^{2})},\;\sigma_{22}=-\frac{\varepsilon_{2}\nu (A,\Phi )\cos^{2}\Phi }{1+K(A)+\alpha A^{2}}.$$

Treating the amplitude A(t) in Equation (18) as a slowly varying process under light damping, weak excitation, and small impact losses, the Stratonovich–Khasminskii theorem [17,18] ensures its asymptotic weak convergence to a diffusion Markov process. The governing Itô equation for the averaged amplitude A(t) is thus obtained:

(20) dA=b(A)dt+σ(A)dB(t).

where the drift b(A) and diffusion coefficients $\sigma^{2}(A)$ have the following explicit expressions:

(21) $$\begin{array}{l} b(A)={\langle m_{1}+\left(\frac{\partial \sigma_{11}}{\partial A}\sigma_{11}+\frac{\partial \sigma_{11}}{\partial \Phi }\sigma_{21}\right)D_{11}+\left(\frac{\partial \sigma_{12}}{\partial A}\sigma_{12}+\frac{\partial \sigma_{12}}{\partial \Phi }\sigma_{22}\right)D_{22}\rangle }_{\Phi },\\ \sigma^{2}(A)={\langle 2D_{11}({\sigma_{11}}^{2}+\sigma_{11}\sigma_{21})+2D_{22}({\sigma_{12}}^{2}+\sigma_{12}\sigma_{22})\rangle }_{\Phi }, \end{array}$$

where ${\langle \cdot \rangle }_{\Phi }$ denotes the time averaging over a quasi-period.

(22) $${\langle \cdot \rangle }_{\Phi }=\frac{1}{2\pi }\int_{0}^{T}\langle \cdot \rangle d\Phi .$$

Substituting Equation (19) into Equation (21) and performing averaging with respect to the final averaged drift and diffusion coefficients yields

(23) $$\begin{array}{c} b(A)=-\frac{1}{\left(1+K(A)+\alpha A^{2}\right)}\left\{\frac{1}{256}c_{3}[16A^{5}(1+K(A))+13\alpha A^{7}]\right.\\ \;\;\left.-\frac{1}{8}c_{2}[A^{3}(1+K(A))+6\alpha A^{5}]-\frac{1}{16}(c_{1}-C(A))[8A(1+K(A))+5\alpha A^{3}]\right\}\\ \;\;-\frac{1}{\pi (1+K(A)+\alpha A^{2})}\left[\left(1-r)A(b_{0}-b_{2}+b_{4}-b_{6})(1+K(A)+(1/2)\alpha A^{2}\right)\right]\\ \;\;+\frac{{\varepsilon_{1}}^{2}D_{11}}{8(1+K(A)+\alpha A^{2})}\left\{(4b_{0}-2b_{4})\times \frac{d}{dA}\left[\frac{b_{0}}{1+K(A)+\alpha A^{2}}\right]+(2b_{2}-2b_{0}-b_{6}\right.)\\ \;\;\times \frac{d}{dA}\left[\frac{b_{2}}{1+K(A)+\alpha A^{2}}\right]+(2b_{4}-b_{2}-b_{6})\times \frac{d}{dA}\left[\frac{b_{4}}{1+K(A)+\alpha A^{2}}\right]+(2b_{6}-b_{4})\\ \;\;\left.\times \frac{d}{dA}\left[\frac{b_{6}}{1+K(A)+\alpha A^{2}}\right]\right\}+\frac{{\varepsilon_{1}}^{2}D_{11}}{8A{\left(1+K(A)+\alpha A^{2}\right)}^{2}}\times [4{b_{0}}^{2}-{b_{2}}^{2}+2{b_{4}}^{2}+2{b_{6}}^{2}]\\ \;\;+\frac{{\varepsilon_{2}}^{2}AD_{22}}{32(1+K(A)+\alpha A^{2})}\left\{\left(2b_{0}-b_{4}\right)\right.\times \frac{d}{dA}\left[\frac{A(2b_{0}-b_{4})}{1+K(A)+\alpha A^{2}}\right]+(b_{2}-b_{6})\\ \;\;\times \frac{d}{dA}\left[\frac{A(b_{2}-b_{6})}{1+K(A)+\alpha A^{2}}\right]+b_{4}\times \frac{d}{dA}\left[\frac{Ab_{4}}{1+K(A)+\alpha A^{2}}\right]+\left.b_{6}\times \frac{d}{dA}\left[\frac{Ab_{6}}{1+K(A)+\alpha A^{2}}\right]\right\}\\ \;\;+\frac{{\varepsilon_{2}}^{2}AD_{22}}{8{\left(1+K(A)+\alpha A^{2}\right)}^{2}}[2{b_{0}}^{2}+2b_{0}b_{2}+{b_{2}}^{2}+b_{2}b_{4}+{b_{4}}^{2}+b_{4}b_{6}+{b_{6}}^{2}], \end{array}$$

(24) $$\sigma^{2}(A)=\frac{{\varepsilon_{1}}^{2}D_{11}(1+K(A)+\frac{5}{8}\alpha A^{2})}{{\left(1+K(A)+\alpha A^{2}\right)}^{2}}+\frac{{\varepsilon_{2}}^{2}A^{2}D_{22}(1+K(A)+\frac{3}{4}\alpha A^{2})}{4{\left(1+K(A)+\alpha A^{2}\right)}^{2}}.$$

## 5. Stationary Response

According to Itô differential Equation (20), the averaged FPK equation has the following form:

(25) $$\frac{\partial }{\partial t}p(A,t)=-\frac{\partial }{\partial A}[b(A)p(A,t)]+\frac{1}{2}\frac{\partial^{2}}{\partial A^{2}}[\sigma^{2}(A)p(A,t)].$$

The corresponding boundary conditions of Equation (25) are as follows:


p=finite   at A=0,

p,  ∂p/∂A→0 at A→∞.

The stationary solution of FPK Equation (25) can be obtained as follows:

(26) $$P(A)=\frac{C}{\sigma^{2}(A)}\exp[\int_{0}^{A}\frac{2b(u)}{\sigma^{2}(u)}du].$$

in which parameter *C* is a normalization constant. Through the stationary probability density with respect to amplitude, the stationary PDF of total energy *H* is derived as

(27) $$p(H)=p(A)\left|\frac{dA}{dH}\right|={\left.\frac{p(A)}{g(A)}\right|}_{A=G^{-1}(H)}.$$

where $G^{-1}$ is the inverse function of G. The joint stationary PDF of transformed variables y and $\dot{y}$ can be evaluated by

$$p(y,\dot{y})={\left.\frac{p(H)}{T(H)}\right|}_{H=(1/2){\dot{y}}^{2}+G(y)},$$

in which

(28) $$T(H)={\left.\frac{2\pi }{\omega (A)}\right|}_{A=G^{-1}(H)}.$$

Utilizing the inverse transformation of formula (12), the stationary united PDF of the original variables x and $\dot{x}$ can be expressed as

(29) $$p(x,\dot{x})=2p_{y,\dot{y}}(x,\dot{x}),x\geq 0.$$

As described by the approximate relation provided in Equation (9) and Equation (29), the MSV of electric voltage can be derived as

(30) $$E[V^{2}]=\int_{-\infty }^{+\infty }\int_{-\infty }^{+\infty }(\frac{\mu }{\mu^{2}+\omega^{2}(A)}x_{2}+\frac{\omega^{2}(A)}{\mu^{2}+\omega^{2}(A)}x_{1}{)}^{2}p(x_{1},x_{2})dx_{2}dx_{2}$$

and the expression of the mean output power is as follows:

(31) $$E[P]=E[P_{V}]=\beta \mu E[V^{2}].$$

## 6. Stochastic Stability

The aim of this section is to analyze the asymptotic stability with probability one in a nonlinear PVEH system under unilateral rigid impact. Random external excitation leads to a limited diffusion of the system’s stationary state, whereas stochastic parametric excitation induces instability, driving the system towards a qualitatively different steady state. Hence, investigating the stability of parametrically excited stochastic vibrations is of greater significance than that of externally excited ones. Therefore, the stochastic stability of a system with only parametric excitation and no external excitation is considered by letting $\varepsilon_{1}=0$. The new system has the following form:

(32a) $$\ddot{x}+(c_{3}x^{4}-c_{2}x^{2}-c_{1})\dot{x}+x+ax^{3}+\beta V=\varepsilon_{2}x\xi_{2}(t),\;x>0,$$

(32b) $${\dot{x}}_{+}=-r{\dot{x}}_{-},\;x=0,$$

(32c) $$\dot{V}+\mu V=\dot{x},$$

Based on the above condition, through linearizing Equation (23) at *A* = 0, we can obtain the corresponding linearized Itô equation:

(33) $$dA=b^{\prime }(0)Adt+\sigma^{\prime }(0)AdB(t),$$

(34) $$b^{\prime }(0)=\frac{c_{1}}{2}-\frac{\beta \mu }{2(\mu^{2}+1+\beta )}-\frac{1}{\pi }(1-r)\sqrt{1+\frac{\beta (1+\beta )}{\mu^{2}+1+\beta }}+\frac{3{\varepsilon_{2}}^{2}D_{22}[1+(\mu^{2}+\beta )+\beta^{2}]}{8(\mu^{2}+1+\beta )},$$

(35) $${\sigma^{\prime }}^{2}(0)=\frac{{\varepsilon_{2}}^{2}D_{22}[1+(\mu^{2}+\beta )+\beta^{2}]}{4(\mu^{2}+1+\beta )}.$$

Introducing the new variable

ρ=lnA.

and applying the Itô differential rule, the corresponding stochastic differential equation for ρ is obtained.

(36) $$d\rho =[b^{\prime }(0)-\frac{1}{2}{\sigma^{\prime }}^{2}(0)]dt+\sigma^{\prime }(0)dB(t).$$

Through integrating Equation (35), one obtains the expression as follows:

(37) $$\rho (t)=\rho (0)+\int_{0}^{t}[b^{\prime }(0)-\frac{1}{2}{\sigma^{\prime }}^{2}(0)]ds+\int_{0}^{t}\sigma^{\prime }(0)dB(s).$$

The Lyapunov exponent of system (33) can be given by the expression below:

$$\begin{array}{c} \lambda =\underset{t\to \infty }{\lim}\frac{1}{t}\ln A=\lim\frac{1}{t}\rho (t)\\ \;\;=\underset{t\to \infty }{\lim}[\frac{\rho (0)}{t}+\frac{1}{t}[b^{\prime }(0)-\frac{1}{2}{\sigma^{\prime }}^{2}(0)]t+\frac{1}{t}\sigma^{\prime }(0)B(t)]\\ \;\;=b^{\prime }(0)-\frac{1}{2}{\sigma^{\prime }}^{2}(0). \end{array}$$

Through substituting Equation (34) and Equation (35) into Equation (38), we can derive the following equation:

(38) $$\lambda =\frac{c_{1}}{2}-\frac{\beta \mu }{2(\mu^{2}+1+\beta )}-\frac{1}{\pi }\left[(1-r)\sqrt{1+\frac{\beta (1+\beta )}{\mu^{2}+1+\beta }}\right]+\frac{{\varepsilon_{2}}^{2}D_{22}[1+\mu^{2}+\beta +\beta^{2}]}{4(\mu^{2}+1+\beta )}.$$

The largest Lyapunov exponent $\lambda_{\max}$ of system (32) is obtained approximately by Equation (38). Moreover, the system (32) is the asymptotic stability with probability one if $\lambda_{\max}<0$, and it is unstable if $\lambda_{\max}>0$.

## 7. Conclusions and Discussion

### 7.1. Validity of the Approach and Analysis of Responses

**Figure 1:**
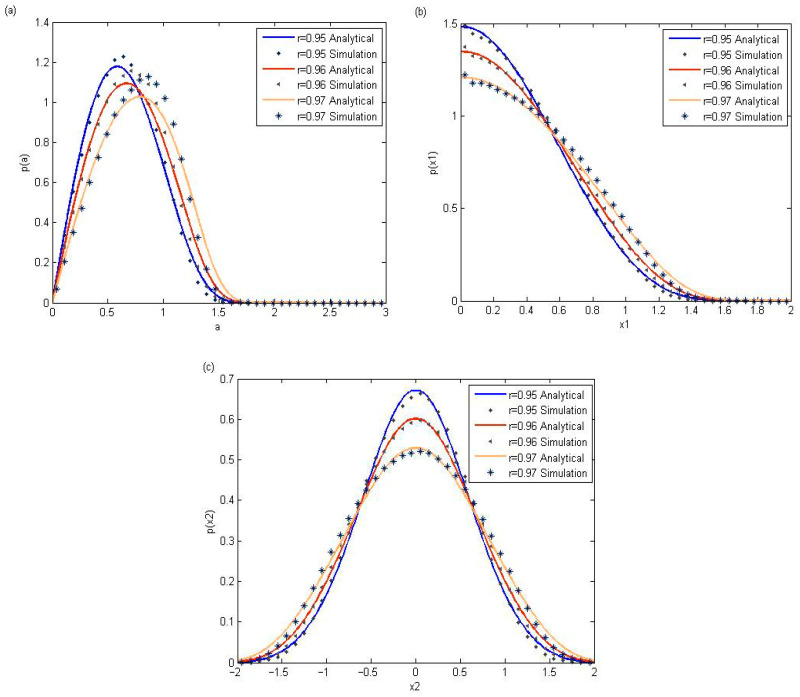
Stationary pdfs with different values of the restitution coefficients *r*, when the parameters $c_{3}=0.1$. (**a**) Probability density of amplitude; (**b**) probability density of displacement; (**c**) probability density of velocity.

**Figure 2:**
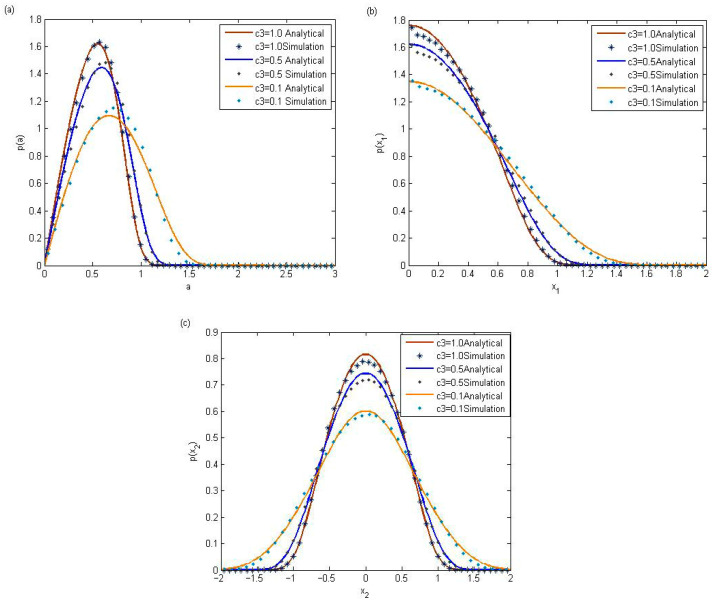
Stationary pdfs with different values of the nonlinear damping coefficients $c_{3}$, when the parameters r=0.96. (**a**) Probability density of amplitude; (**b**) probability density of displacement; (**c**) probability density of velocity.

In order to verify the effectiveness of the above proposed analytical method, Monte Carlo simulations are adopted to compare with the theoretical results in Figure 1 and Figure 2 in this section. The fourth-order Runge–Kutta algorithm is utilized to obtain the numerical results. During the Monte Carlo simulation process, 80 × 80 initial points are selected, and 1000 random trajectories are generated from each point. The system parameters $\omega_{0}=1.0,\;\alpha =0.5,$ β=0.05, $\mu =0.05,\;c_{2}=0.05,$ $c_{1}=0.01,$ $D_{11}=0.01,$ $D_{22}=0.01$ are fixed to investigate the effects of the restitution coefficient and nonlinear damping coefficient. Figure 1 shows the effect of different restitution coefficients on the stationary probability density functions. It is obvious that the reduction in the restitution coefficient can lead to the higher peak value of probability density functions.

To illustrate their dependence on the nonlinear damping coefficient $c_{3}$, Figure 2 displays the stationary probability density functions corresponding to amplitude, displacement, and velocity. An increase in the nonlinear damping coefficient is associated with a corresponding rise in the peak values of the probability density functions. Figure 1 and Figure 2 indicate the availability of the theoretical methods which are adopted in the above section for the stochastic PVEH system with impact.

### 7.2. Influence on the MSV

**Figure 3:**
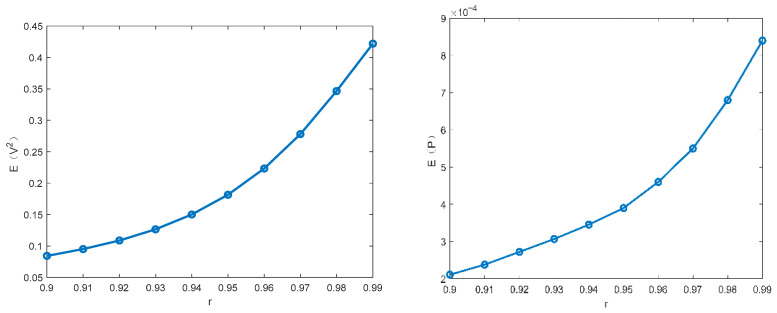
Influence of restitution coefficient r on the MSV $E(V^{2})$ and the mean output power E(P).

**Figure 4:**
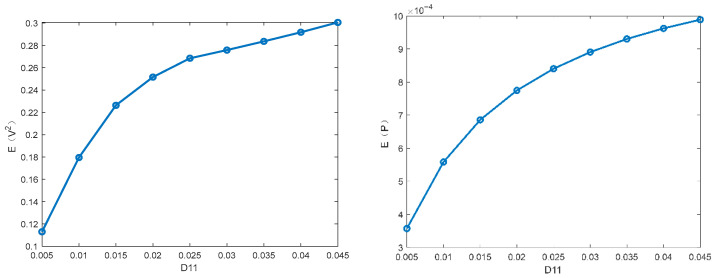
Influence of noise intensity $D_{11}$ on the MSV $E(V^{2})$ and the mean output power E(P).

This subsection focuses on discussing the influence of different parameters on the MSV and mean output power of the PVEH system via the proposed technique. The parameters $\omega_{0}=1.0,\alpha =0.5,$ $c_{3}=0.1,$ $c_{2}=0.05,$ $c_{1}=0.01,$ $D_{22}=0.01$ are fixed in the following figures. Figure 3 and Figure 4 illustrate how the MSV and mean output power depend on the restitution coefficient and the white noise intensity. The MSV increases significantly with the restitution coefficient, suggesting that the impact structure enhances the harvester’s energy output. It can be seen that the MSV almost increases proportionally with the excitation intensity. Furthermore, it is evident that the trend in mean output power closely mirrors that of the MSV.

**Figure 5:**
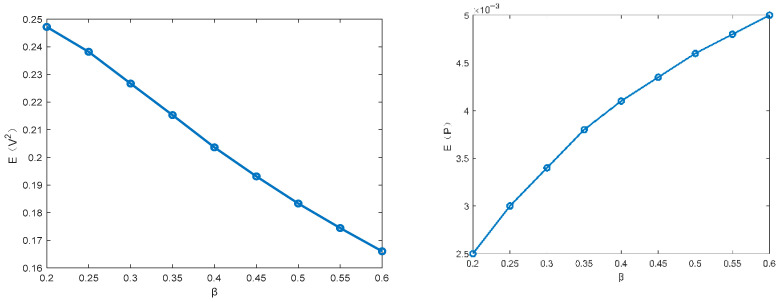
Influence of the time constant ratio β on the mean square voltage $E(V^{2})$ and the mean output power E(P).

**Figure 6:**
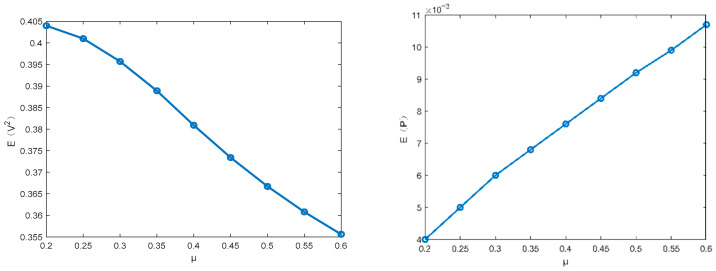
Influence of the time constant ratio μ on the MSV $E(V^{2})$ and the mean output power E(P).

Figure 5 shows that the mean square voltage (MSV) decreases with an increasing piezoelectric coupling coefficient β, while a parallel trend is observed in Figure 6 for the influence of the time constant ratio μ. Furthermore, because the mean output power depends on the MSV, their trends are opposed—a finding that is consistent with the conclusion provided by Equation (31). Based on the above analysis, the changes in the piezoelectric coupling coefficient β, the time constant ratio μ, the restitution coefficient and the excitation intensity can improve the harvest performance.

### 7.3. Discussion of Stochastic Stability

**Figure 7:**
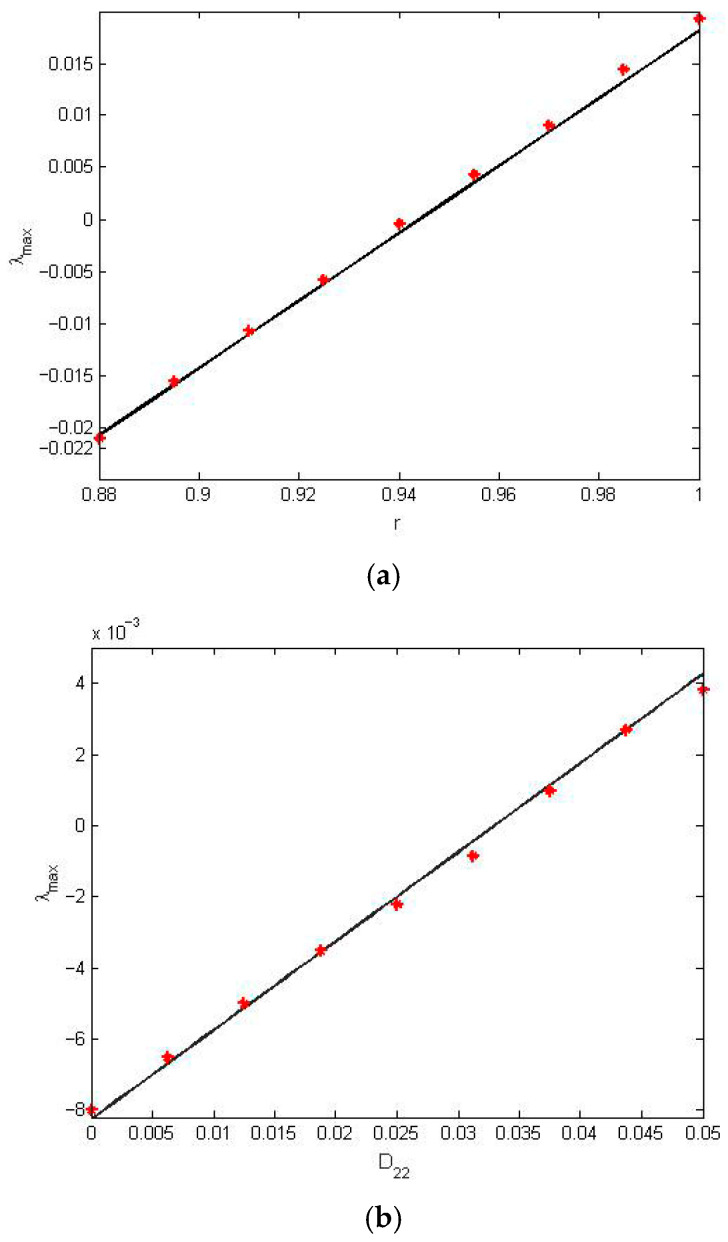
(**a**) Effects of the restitution coefficient r on the TLE $\lambda_{\max}$. (**b**) Effects of the noise intensity $D_{22}$ on the TLE $\lambda_{\max}$. (−) Analytical results; (*) numerical results.

**Figure 8:**
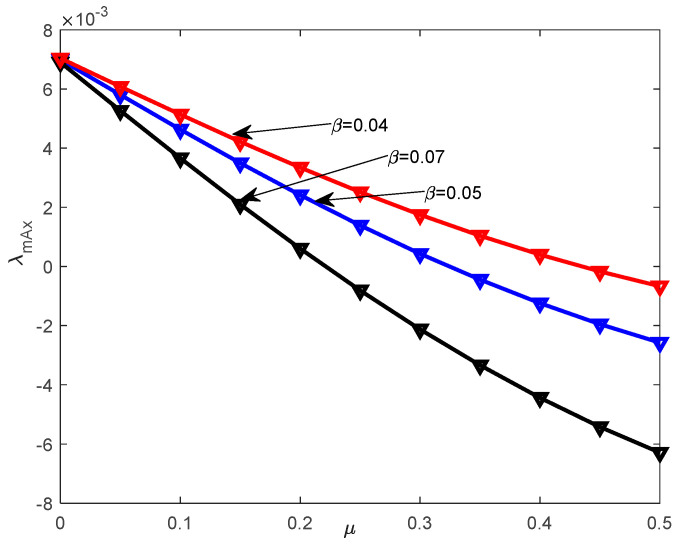
Effects of the different time constant ratio μ on the TLE $\lambda_{\max}$.

**Figure 9:**
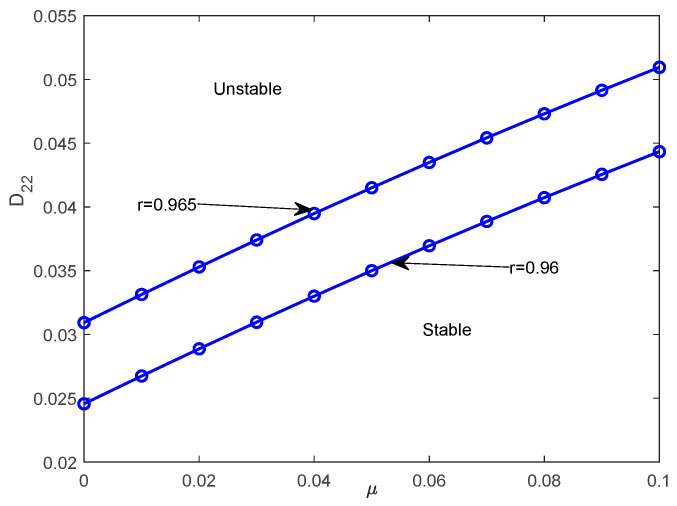
Region of asymptotic Lyapunov stability with probability one in the plane $\left(\mu ,D_{22}\right)$ for different restitution coefficients r.

The objective of this subsection is to investigate the stochastic asymptotic stability with probability one for system (32). This analysis is conducted based on the top Lyapunov exponent (TLE) obtained in Section 6. The effects of the restitution coefficient and noise intensity on the stability of the system are investigated. The parameters $c_{3}=0.025,$ $c_{2}=0.015,$ $c_{1}=0.01,$ α=0.05, β=0.04, μ=0.1, $\omega_{0}=1.0,\;\varepsilon_{1}=0$ are fixed. Figure 7a illustrates the effects of the restitution coefficient r on the TLE $\lambda_{\max}$, where $D_{22}=0.06$. The TLE $\lambda_{\max}$ increases monotonically with the parameter r. Correspondingly, the system’s stability state undergoes a transition as r increases. Figure 7b describes the variation in the noise intensity $D_{22}$ on the TLE $\lambda_{\max}$ when assuming that r=0.96. It can be obviously seen that the TLE $\lambda_{\max}$ increases along with the increase in $D_{22}$. From Figure 7a,b, it can be concluded that the analytical results obtained from the proposed method agree with the results from the digital simulation, which indicates that the analytical method proposed in Section 3 is effective. The TLE $\lambda_{\max}$ for different time constant ratios μ and nonlinear stiffness coefficients β are shown in Figure 8. The TLE decreases gradually with the increase in the time constant ratio μ and nonlinear stiffness coefficient β. Figure 8 indicates that an increase in the time constant ratio μ corresponds to improved stability for system (32). Figure 9 depicts the boundary of asymptotic stability with probability one with different restitution coefficients r on the stability region in the plane $\left(\mu ,D_{22}\right)$. It can be concluded that the stable region increases as the restitution coefficient r decreases.

## 8. Conclusions

This study presents an analytical investigation into the stochastic response and stability of a nonlinear piezoelectric energy harvester featuring a unilateral offset barrier. An equivalent nonlinear system is first derived via generalized harmonic transformation. Subsequently, the stochastic averaging method, grounded in generalized harmonic functions, is employed to derive the averaged Itô stochastic differential equation for the modified system. Numerical simulations are conducted to validate the proposed analytical approach. The influence of key system parameters on the mean square value (MSV) and the mean output power is examined. Furthermore, the expression for the largest Lyapunov exponent (TLE) is derived to assess the system’s asymptotic stability with probability one. The analysis demonstrates that system stability can be effectively modulated by adjusting the restitution coefficient, noise intensity, time constant ratio, and nonlinear stiffness coefficient.

## Data Availability

No data was used for the research described in the article.
